# Factors Affecting the Success Rate of Percutaneous Nephrolithotomy in Paediatric Patients

**DOI:** 10.3390/jcm7030043

**Published:** 2018-03-04

**Authors:** Hikmat Jabrayilov, Murat Yavuz Koparal, Serhat Gürocak, Bora Küpeli, Mustafa Özgür Tan

**Affiliations:** 1 Urology Clinic, Medical Park Ankara Hospital, Ankara 06370 Turkey; dr.hikmetc@gmail.com; 2 Department of Urology, Recep Tayyip Erdogan University Training and Research Hospital, Rize 53020, Turkey; 3 Department of Urology, School of Medicine, Gazi University, Ankara 06500, Turkey; sgurocakmd@yahoo.com (S.G.); borakupeli@yahoo.com (B.K.); mozgurtan@yahoo.com (M.Ö.T.)

**Keywords:** percutaneous nephrolithotomy, urolithiasis, paediatric, stone

## Abstract

In this study, we aimed to determine factors affecting the success rate of percutaneous nephrolithotomy (PNL) in children. The series consisted of 41 consecutive children operated on by the same surgical team for renal calculi with PNL between June 2002 and May 2015 in our institution. A single calyx or pelvic stone was described as simple, while calculi located in more than one location (calyx and pelvis or more than one calices) or staghorn stones were described as complex. The procedure was deemed successful if the patient was completely stone-free (SF) or had residual fragments <4 mm. Thirty-four patients were found to be SF or had residual fragments <4 mm on the postoperative first day, thus the success rate was 82.9%. In complex stones, the success rate was significantly lower (45.5%) than simple stones (96.7%) (*p* < 0.001). The grade of hydronephrosis (Grade 0–1 vs. Grade 2–3) also had a negative impact on the success, with rates of 92.6% vs. 64.3%, respectively (*p* = 0.022). Previous urological procedure history on the same side yielded a success rate of 58.3%, whereas the success rate in the primary patients was 93.1% (*p* < 0.001). The localization of the stone (complex vs. simple), degree of hydronephrosis, and history of previous urological procedures were found to be the factors that affected the success of the paediatric PNL.

## 1. Introduction

Predisposing factors, such as metabolic disorders, infections, and congenital anomalies, are more common in paediatric patients with urinary calculi; therefore, the expected stone recurrence rates are higher than adults, demanding successful treatment using minimally invasive methods [1]. Similar to adults, usual recommendation as the first line therapy for the treatment of kidney stones smaller than 2 cm (except lower caliceal calculi due to lower rates of clearance) is extracorporeal shock wave lithotripsy (ESWL) [2,3,4,5]. Endoscopic procedures, mainly percutaneous nephrolithotomy (PNL), are valid alternatives to ESWL, with higher stone free rates and a reduced number of repeated treatments. The main indications for PNL are large (high stone burden) and complex stones (more than one location or staghorn), severe kidney obstruction (high grade hydronephrosis), hard stones (cystine), or ESWL failures [6,7]. In parallel with technological progress, endoscopic instruments have become thinner and are of a higher quality, like Mini-perc and Micro-perc, providing even less invasive and successful treatment of stone disease in children. The stone-free (SF) rates after PNL in children vary greatly between 73% and 96% depending on the stone burden and the so-called term complex calculi (usually defined as staghorn, more than one location, anomalous kidneys, and so on) [6,7]. In this respect, we analysed factors affecting the success rate of PNL in children.

## 2. Patients and Method

The series consisted of 41 consecutive children, 20 (48.8%) girls and 21 (51.2%) boys, operated on by the same surgical team for renal calculi by PNL between June 2002 and May 2015 in our institution. The indications of PNL were a high stone burden (>2 cm in diameter, staghorn calculi), failure of previous treatment (ESWL and so on), cystine calculi, and severe hydronephrosis-necessity of quick treatment. The patient characteristics (age, sex, body mass index (BMI), and concurrent disease history), radiological diagnostic methods, stone and urinary system characteristics (side, stone burden, localization, grade of hydronephrosis, and previous urological procedure history), operation parameters (location of access, diameter of the sheath, presence of complications, duration of anaesthesia, and fluoroscopy), and postoperative data (SF status, catheter use and duration of catheterization, and hospitalization) were evaluated as possible factors affecting the success of the PNL.

### 2.1. Preoperative Evaluation

Detailed medical histories were obtained from each patient (including any systemic diseases) and physical examinations were performed. Preoperative radiological evaluation was performed using ultrasonography (USG), plain abdominal X-ray, intravenous urography (IVU), and unenhanced spiral computed tomography (sCT). The grade of hydronephrosis was determined according to the Society for Fetal Urology grading scale, and the patients were divided into two groups as low grade (0–1) and high grade (2–3) [8]. A single calyx or pelvic stone was described as simple, while calculi located in more than one location (calyx and pelvis or more than one calices) or staghorn stones were described as complex [9]. The stone burden was calculated by multiplying the long axis diameter by the diameter perpendicular to it [10]. In the case of multiple stones, the total value after the summation of independent calculi was accepted as the stone burden. The modified Clavien classification was used to rate the perioperative complications [11].

### 2.2. Surgical Technique

All of the patients underwent standard PNL procedure under general anaesthesia. Initially, a 5 Fr open-ended ureteral catheter was placed endoscopically. Then, the patient was turned to a prone position and contrast material was injected through the ureteral catheter for opacification of the pyelo-calyceal system. Appropriate calyceal entry was done with the needle using the usual technique under fluoroscopic guidance. Dilation of the nephrostomy access was obtained by Amplatz dilators up to a maximum of 24 Fr. The calculi were fragmented by pneumatic or laser lithotripsy and the stone pieces were extruded with forceps usually with a 17 Fr nephroscope (Karl Storz^®^, Tuttlingen, Germany). The procedure was terminated by placing an appropriate size nephrostomy catheter.

### 2.3. Postoperative Evaluation

The success of the operation was determined using abdominal X-ray on the first postoperative day. The procedure was deemed successful if the patient was completely stone-free (SF) or had residual fragments <4 mm. The urethral catheter was removed on the first postoperative day. An antegrade nephrostogram was performed on the second postoperative day, and the nephrostomy catheter was withdrawn in patients with a through passage of fluid into the bladder. In the presence of obstruction or percutaneous urinary leak that lasted longer than 24 h, a double J (DJ) catheter was inserted.

### 2.4. Statistical Evaluation

Statistical Package for the Social Sciences-15 (SPSS-15, Chicago, IL, USA) was used for the statistical analysis. The chi-squared and Fisher’s exact tests were used, and *p* < 0.05 was considered to be statistically significant.

## 3. Results

The mean age at the time of surgery was 9.9 ± 4.89 (2–17) years and the mean BMI was 19.89 ± 3.96 kg/m^2^. Preoperatively, six of the 41 patients were diagnosed with USG and X-ray, 19 were diagnosed with USG and IVU, 11 were diagnosed with IVU only, and five were diagnosed with USG and sCT (Table 1). A lower calyceal entry was performed in 19 cases (46.3%), while a middle calyceal entry was performed in 22 (53.7%). Thus, one working channel was created with a subcostal entry in all of the patients. The operation parameters are given in Table 2.

The mean stone burden was calculated as 6.34 ± 2.05 (1.9–9.7) cm^2^. For easy analysis, patients were grouped in terms of their stone burden, as <5 cm^2^ (10 patients) and ≥5 cm^2^ (31 patients). Thirty-four patients were found to be SF or had residual fragments <4 mm on the postoperative first day, thus the success rate was 82.9%. Apart from five patients with cystinosis, most patients had calcium oxalate stones. The size and success rates according to stone localization are given at Table 3. There was no statistically significant difference between age groups (*p* = 0.777) and stone burden groups (<5 cm^2^ vs. ≥5 cm^2^) (*p* = 0.4111) for the success rate. However, in complex stones, the success rate was significantly lower (45.5%) than simple stones (96.7%) (*p* < 0.001). The grade of hydronephrosis (Grade 0–1 vs. Grade 2–3) also had a negative impact on the success, with rates of 92.6% vs. 64.3%, respectively (*p* = 0.022). Previous urological procedure history on the same side yielded a success rate of 58.3%, whereas the success rate in the primary patients was 93.1% (*p* < 0.001). Excluding the complex stones, the effect of previous intervention on the success rate was also analysed in the simple stone group. We found that previous intervention (retrograde intrarenal surgery particularly in this group) was also associated with a lower success rate (*p* = 0.007). There were no statistically significant relationships between the success rate and the BMI, access site, sheath size, or complications (*p* > 0.05) (Table 4).

Complications occurred in nine (22%) patients. Some patients have more than one complication but most were minor (Table 5). A DJ catheter was inserted in two patients due to urine leakage (grade 3b complication) for longer than 24 h and in one patient due to a ureteral stone obstruction (grade 3b complication). None of the patients had grade 4 or 5 complications.

## 4. Discussion

The success and complication rates of PNL in experienced hands in the paediatric age group are similar to adults. In children, the efficacy and reliability of PNL has been shown in many studies, with high but variable (73–96%) SF rates. This wide range may be due to differences in stone size and renal anatomy, as well as changing body image with age [6,7,12,13,14]. In 1985, Segura et al. reported a 98% success rate in a total of PNL 1000 cases, which was the first study with a large series of patients [15]. In our study, a success rate of 82.9% was obtained in 41 children undergoing PNL with an SF rate of 53.7%, and 29.2% of the children had residual fragments <4 mm. Though the initial SF rate seems low, it was the evaluation at the postoperative first day. The determination of success rate by an early X-ray is of course an initial measure but it is very useful. The ideal reliable method would be the use of computed tomography (CTscan), but due to the radiation exposure, ultrasound is the mainstay of follow up in children. The stone burden was also high in our series, with a value of 6.34 ± 2.05 (1.9–9.7) cm^2^, and our clinic is a referral center. Thus, we had high rates of complex stones, and we treated treatment failures and complicated cases. About half of our patients had accompanying diseases like cytinosis, infection, and metabolic disorders that are highly related with stone formation and recurrence. Additionally, previous unsuccessful treatment might cause residual fragments that would provide nidus for recurrent complex calculi.

Factors affecting PNL success rates were examined by Goldwasser et al. and the authors reported that the size of the stone and its localization mostly affected the success rates [16]. Consistent with the literature, in terms of stone localization, we also found that the success rate in complex stones was lower than that in simple stones [7,17,18,19,20]. Our success rate in simple stones was 96.7% (single stone in pelvis or a calyx), but 45.5% in complex stones (*p* < 0.001). Partial staghorn stones were better, with a value of 75% in terms of success. European Association of Urology (EAU) guidelines offer PNL as the first treatment option for staghorn stones [21]. We thought that multiple working channels could increase the complication rates, so we created a single subcostal channel for all of our patients. The reported success rates in complex stones ranged from 61% to 89% with the use of multiple channels [7,17,18,19,20]. Netto et al. reported that the success of percutaneous treatment for staghorn calculi is highly related to optimal kidney access with supracostal and multiple access approaches, despite a slight increase in the incidence of acceptable complications [22]. However, Ozden et al. reported an SF rate of 74% using a single channel in 60% of the children with complex stones. Increasing the number of tracts was significantly related with more blood loss, increasing the complication rate [7]. As expected, the stone burden was higher in patients who needed multiple tracts [7]. Thus, experience and stone characteristics, mainly burden and complexity, appear to be the main determinants for the number of tracts. As stated before, the main reason for our low success rate in complex renal calculi was our avoidance of multiple channel use due to less experience in the paediatric age group. Increasing experience with the use of multiple tracts could achieve better stone-free rates.

In hydronephrotic systems with stones, the entrance is easy, but the rate of failure increases because the stones can escape to other calyces [23]. In our study, 25 of the 34 successful paediatric PNL procedures (73.5%) had lower grades of hydronephrosis. In this patient group, more than one working channel could be used when the stones could not be reached from the entrance tract like in complex stones.

History of surgery and/or ESWL was found to be a statistically significant factor affecting the success rate in paediatric PNL in our study. The PNL procedure failed in five out of 12 patients with prior histories of surgery or ESWL (41.7%). Basiri et al. stated that an open surgical history on the same side did not affect the success or complication rates in patients treated with PNL [24]. Yuruk et al. also reported that similar success and complication rates could be achieved with PNL after failed ESWL, but it was usually more difficult with a prolonged operative time and fluoroscopic screening time [25]. In our study, seven of our 12 patients with a same side urological procedure history had complex stones which might be the reason for the decreased surgical success. However, we performed another analysis in the simple calculi group (which has more number of patients) to see the effect of previous surgery on the success rate Excluding the complex stones, the effect of previous intervention on the success rate was also analysed in the simple stone group. We found that previous intervention (retrograde intrarenal surgery particularly in this group) was also associated with a lower success rate (*p* = 0.007). However, as noted before, previous unsuccessful treatment might result in residual fragments scattered all over kidney that would be difficult to extract with a single tract or provide nidus for further stone formation at multiple sites rather than technical problems.

The experience of the surgeon is an important factor affecting the overall surgical success. Evaluating the learning curve in PNL, Allen et al. emphasized that the surgeon could achieve adequacy after 60 operations and excellence after 115 operations [26]. Onal et al. recommended that surgeons should complete at least 100 cases in the adult patient group to gain experience before attempting this procedure in the paediatric patient group [27]. Song et al. found that there was a statistically significant difference between the first 15 cases and the 45th through 60th cases in terms of the mean screening time and duration of the tract dilatation. There were no significant differences between the groups in terms of the SF and complication rates [28].

Retrograde intrarenal surgery is performed in patients with a high renal stone burden, but the guidelines still do not offer it as the first line treatment. There is very limited experience in the paediatric age group and lower stone-free rates compared to PNL [29].

A major limitation of our study is the low number of patients. However, it yields useful information by a single surgical team experience in a referral center treating complex cases. The team has experience in adult PNL, thus the success rate is acceptable for the series. Increasing experience and use of multiple access would provide better results. Though we have acceptable complication rates, newer techniques like Micro-perc would serve as even less invasive treatment strategies.

## 5. Conclusions

Overall localization of the stone (complex vs. simple), degree of hydronephrosis, and history of previous urological procedures affected the success of the paediatric PNL.

## Figures and Tables

**Table 1:** Clinical features of patients.

		*n* (%)
Side	Right	29 (70.7)
	Left	12 (29.3)
Same side previous urological procedure history	Extracorporeal shock wave lithotripsy	6 (14.6)
	Ureterorenoscopy	3 (7.3)
	Retrograde intrarenal surgery	2 (4.9)
	Pyelolithotomy	7 (17.1)
	No previous procedure	23 (56.1)
Grade of hydronephrosis	Grade 0	19 (46.3)
	Grade 1	8 (19.5)
	Grade 2	10 (24.4)
	Grade 3	4 (9.8)
Radiological diagnostic method	Abdominal X-ray and US	6 (14.6)
	Intravenous urography and US	19 (46.3)
	Intravenous urography	11 (26.8)
	US and sCT	5 (12.2)
Accompanying disease	Frequent urinary tract infection	7 (17.1)
	Contralateral stone	5 (12.2)
	Cystinosis	5 (12.2)
	Vesicoureteral reflux	1 (2.4)
	Tip 1 Hypercalciuria	1 (2.4)
	Chronic renal disease	1 (2.4)
	Osteogenesis imperfecta	1 (2.4)
	No accompanying disease	20 (48.8)

Abbreviations. SD: Standart deviation, US: Ultrasonography, sCT: Unenhanced spiral computed tomography.

**Table 2:** Operation and hospitalization parameters of patients.

	Mean (Min–Max)
Duration of anaesthesia (minute)	122.44 (75–195)
Duration of fluoroscopy (minute)	2.53 (0.5–10.0)
Amount of irrigation (liter)	5.01 (2.2–9.0)
Duration of operation (minute)	102.07 (60–180)
Duration of diversion (day)	2.45 (1–5)
Duration of postoperative hospitalization (day)	4.27 (2–12)
Duration of total hospitalization (day)	7.41 (2–18)

**Table 3:** Size and success rates of stones according to localizations.

	Overall *n* (%)	Mean Stone Burden (cm^2^) ± SS	Successful		Unsuccessful *n* (%)
			Stone Free *n* (%)	<4 mm Residual Fragment *n* (%)	
Simple Stones	30 (73.2)	5.89 ± 1.99	20 (66.7)	9 (30)	1 (3.3)
Isolated renal pelvic	18 (43.9)	6.08	14 (77.8)	3 (16.7)	1 (5.6)
Isolated lower calyceal	6 (14.6)	5.84	4 (66.7)	2 (33.3)	0
Isolated medium calyceal	1 (2.4)	3.60	0	1 (100)	0
Isolated upper calyceal	5 (12.2)	6.0	2 (40)	3 (60)	0
Complex Stones	11 (26.8)	7.57 ± 1.73	4 (36.4)	1 (9.1)	6 (54.5)
Staghorn	2 (4.9)	7.35	1 (50)	0	1 (50)
Partial staghorn	4 (9.8)	8.88	3 (75)	1 (25)	0
Renal pelvic and lower calyceal	3 (7.3)	6.87	0	0	3 (100)
Medium and upper calyceal	1 (2.4)	4.50	0	0	1 (100)
Multiple calyceal	1 (2.4)	8.0	0	0	1 (100)
Overall	41	6.34 ± 2.05	22 (53.7)	12 (29.3)	7 (17.1)

**Table 4:** Factors affecting the success of percutaneous nephrolithotomy.

		Successful *n* (%)	Unsuccessful *n* (%)	*p*-Value
Age (year)	<5	6 (75)	2 (25)	0.777
	5–12	15 (83.3)	3 (16.7)	
	13–18	13 (86.7)	2 (13.3)	
Body mass index (kg/m^2^)	<25	28 (82.4)	6 (17.6)	0.830
	≥25	6 (85.7)	1 (14.3)	
Stone burden (cm^2^)	<5	10 (90.9)	1 (9.1)	0.411
	≥5	24 (80)	6 (20)	
Stone localization	Simple	29 (96.7)	1 (3.3)	<0.001
	Complex	5 (45.5)	6 (54.5)	
Grade of hydronephrosis	Gr 0–1	25 (92.6)	2 (7.4)	0.022
	Gr 2–4	9 (64.3)	5 (35.7)	
Location of access	Lower calyx	16 (84.2)	3 (15.8)	0.839
	Medium calyx	18 (81.8)	4 (18.2)	
Diameter of sheath (Fr)	<20	29 (85.3)	5 (14.7)	0.375
	≥20	5 (71.4)	2 (28.6)	
Complications	No	28 (87.5)	4 (12.5)	0.142
	Yes	6 (66.7)	3 (33.3)	
Same side previous urological procedure history	No	27 (93.1)	2 (6.9)	<0.001
	Yes	7 (58.3)	5 (41.7)	

**Table 5:** Complications according to modified Clavien classification.

Grade	*n* (%)
Grade 1	8 (44.4)
Fever	4 (22.2)
Transient serum creatinine elevation	3 (16.6)
Wound infection	1 (5.6)
Grade 2	7 (38.8)
Blood transfusion	3 (16.6)
Urinary tract infection	4 (22.2)
Grade 3a	2 (11.1)
Urine leakage for longer than 24 hours	2 (11.1)
Grade 3b	1 (5.6)
Ureteral obstruction related to stone	1 (5.6)
Grade 4a	0
Grade 4b	0
Grade 5	0
